# Non-Singular Finite Time Tracking Control Approach Based on Disturbance Observers for Perturbed Quadrotor Unmanned Aerial Vehicles

**DOI:** 10.3390/s22072785

**Published:** 2022-04-05

**Authors:** Fayez F. M. El-Sousy, Khalid A. Alattas, Omid Mofid, Saleh Mobayen, Jihad H. Asad, Paweł Skruch, Wudhichai Assawinchaichote

**Affiliations:** 1 Department of Electrical Engineering, Prince Sattam Bin Abdulaziz University, Al Kharj 11942, Saudi Arabia; f.elsousy@psau.edu.sa; 2 Department of Computer Science and Artificial Intelligence, College of Computer Science and Engineering, University of Jeddah, Jeddah 21959, Saudi Arabia; kaalattas@uj.edu.sa; 3 Future Technology Research Center, National Yunlin University of Science and Technology, Douliu 64002, Taiwan; d10913003@yuntech.edu.tw; 4 Department of Physics, Faculty of Applied Sciences, Palestine Technical University, Kadoorie, Tulkarm P.O. Box 7, Palestine; j.asad@ptuk.edu.ps; 5 Department of Automatic Control and Robotics, AGH University of Science and Technology, 30-059 Kraków, Poland; pawel.skruch@agh.edu.pl; 6 Department of Electronic and Telecommunication Engineering, Faculty of Engineering, King Mongkut’s University of Technology Thonburi, Bangkok 10140, Thailand

**Keywords:** quadrotor unmanned aerial vehicle, disturbance observer, non-singular terminal sliding mode, finite-time convergence, wind perturbation

## Abstract

In this paper, a disturbance observer based on the non-singular terminal sliding mode control method was presented for the quadrotor in the presence of wind perturbation. First, the position and attitude dynamical equation of the quadrotor was introduced in the existence of windy perturbation. It was difficult to exactly determine the upper bound of the perturbations in the practical systems such as robot manipulators and quadrotor UAVs. Then, a disturbance observer was designed for the estimation of wind perturbation which was entered to the quadrotor system at any moment. Afterward, a non-singular terminal sliding surface was proposed based on the disturbance observer variable. Furthermore, finite time convergence of the closed-loop position and attitude models of the quadrotor was proved using Lyapunov theory concept. Unlike the existing methods, the new adaptive non-singular terminal sliding mode tracker for quadrotor unmanned aerial vehicles enabled accurate tracking control, robust performance, and parameter tuning. Through the combination of the finite time tracker and disturbance observer, the position and attitude tracking of quadrotor UAVs could be accurately performed not only in the nominal environment but also in the existence of different types of perturbations. Finally, simulation results based on the recommended method were provided to validate the proficiency of the suggested method. Moreover, comparison results with another existing study were presented to prove the success of the proposed method.

## 1. Introduction

Nowadays, quadrotors or unmanned aerial vehicles (UAVs) have attracted more interest due to their favorable properties such as small size and low cost [1,2,3]. These characteristics of quadrotors present challenges in controlling them [4,5]. Moreover, in the control process of quadrotor UAV, investigation of robustness against exterior perturbations such as wind disturbance and sensor failure are counted as a major part of control strategy [6,7]. Hence, with the utilization of a disturbance observer, disturbance can be observed and suppressed immediately, which leads to improvement of performance of the quadrotor system [8,9]. Therefore, an advanced control strategy for quadrotor can be combined with the disturbance rejection technique [10,11,12].

In [13], for reduction in the complexity of the control design, a dynamical model of a perturbed quadrotor was decomposed into two different subsystems which could be controlled independently. Then, the presented nonlinear disturbance observer based on the backstepping control scheme was designed for the first subsystem while the multivariable sliding mode control (SMC) was presented for the second subsystem. In [14], an adaptive neural-discrete time control method based on the fractional-order technique was presented for the quadrotor in the presence of external disturbances. Additionally, for the rejection of disturbance, a discrete disturbance observer was proposed to approximate the external disturbance. In [15], a disturbance observer based on the linear quadratic regulator (LQR) method was presented for the quadrotors under external disturbances and input saturations. Moreover, an anti-wind-up scheme was introduced to tackle the input saturation. In [16], a disturbance observer based on the backstepping control method was proposed for the control of quadrotor in the presence of exterior disturbance. Afterward, in order to enhance the transient and steady-state responses of the control method, the prescribed performance function was introduced. In [17], a nonlinear backstepping control method for tracking control of quadrotor in the appearance of external disturbance was offered. Afterward, to reject the disturbance, a disturbance observer was designed in [17]. In [18], an adaptive feedback control scheme for stabilization of the quadrotor under parameter uncertainty and external disturbance was suggested. Then, a disturbance observer based on the barrier function was planned to estimate the external disturbance. In [19], the external disturbance related to the atmospheric condition was modelled at first. Then, a robust fractional-order SMC technique was presented for tracking control of quadrotor. In [20], stabilization of quadrotor in the presence of external disturbance was examined. Furthermore, an active disturbance observer was presented with the aim of disturbance rejection. Another challenge in the control of the quadrotor is the position and attitude tracking control of the quadrotor with fast convergence. Hence, the non-singular terminal sliding mode control (TSMC) method was adopted with the target of acceleration of reachability of trajectories of the position and attitude of the quadrotor system [21,22,23,24,25,26,27]. In [28], non-singular TSMC was designed for the tracking control of quadrotor. In [29], a non-singular fast terminal sliding mode control (FTSMC) based on the time-varying formation tracking was recommended for quadrotor under external perturbation. In [30], attitude and position tracking control of quadrotor in the presence of exterior disturbance was investigated. Hence, high-order SMC disturbance observer was presented in order to estimate disturbance. Then, the composite non-singular TSMC method was offered for the tracking control [31]. In [32], a non-singular TSMC was suggested for the attitude control of the quadrotor in the existence of fault tolerant and exterior disturbance. Furthermore, an observer based on the neural network was adopted for approximation of the fault tolerant and external perturbation.

According to the review of above-cited papers, there are few comprehensive studies that have investigated the position and attitude tracking control of quadrotor based on the non-singular TSMC method using a disturbance observer. For this reason, a new disturbance observer based on the non-singular TSMC method was proposed with the aim of position and attitude tracking control of quadrotor UAV in the appearance of wind perturbation. For easy perception of the innovation of this study, the basic novelties are summarized below:

- Design of a new disturbance observer combined with non-singular terminal sliding mode control for approximation of wind perturbation;
- Proposition of a non-singular terminal sliding surface with fast convergence rate for position and attitude tracking control of quadrotors; and
- Finite time reachability of the proposed sliding surface based on the Lyapunov stability theory.

For better reading of this paper, the next sections are listed as follows: in Section 2, the position and attitude dynamic models of quadrotor are introduced under wind perturbation. In Section 3, the disturbance observer design is presented. In Section 4, the non-singular TSMC method is reported. Simulation results are displayed in Section 5. Finally, the conclusion is stated in Section 6.

## 2. Model Description of Quadrotor and Some Preliminaries

The under-actuated dynamical model of quadrotor is expressed as [33]:

(1) $$\ddot{x}\left(t\right)=\frac{1}{m}\left[-K_{fdx}\dot{x}+\left(C_{\phi }S_{\theta }C_{\psi }+S_{\phi }S_{\psi }\right)u_{z}\right]\;\ddot{y}\left(t\right)=\frac{1}{m}\left[-K_{fdy}\dot{y}+\left(C_{\phi }S_{\theta }S_{\psi }-S_{\phi }C_{\psi }\right)u_{z}\right]\;\ddot{z}\left(t\right)=\frac{1}{m}\left[-K_{fdz}\dot{z}+\left(C_{\phi }C_{\theta }\right)u_{z}\right]-g\;\ddot{\phi }\left(t\right)=\frac{1}{I_{x}}\left[\left(I_{y}-I_{z}\right)\dot{\psi }\dot{\theta }-K_{fax}{\dot{\phi }}^{2}-J_{r}\bar{\Omega }\dot{\theta }+du_{\phi }\right]\;\ddot{\theta }\left(t\right)=\frac{1}{I_{y}}\left[\left(I_{z}-I_{x}\right)\dot{\psi }\dot{\phi }-K_{fay}{\dot{\theta }}^{2}+J_{r}\bar{\Omega }\dot{\phi }+du_{\theta }\right]\;\ddot{\psi }\left(t\right)=\frac{1}{I_{z}}\left[\left(I_{x}-I_{y}\right)\dot{\phi }\dot{\theta }-K_{faz}{\dot{\psi }}^{2}+C_{D}u_{\psi }\right]$$

where by definition of $u_{x}\left(t\right)=C_{\phi }S_{\theta }C_{\psi }+S_{\phi }S_{\psi }$ and $u_{y}\left(t\right)=C_{\phi }S_{\theta }S_{\psi }-S_{\phi }C_{\psi }$ as supplementary control inputs, the dynamical model of the quadrotor is considered as:

(2) $$\ddot{x}\left(t\right)=\frac{1}{m}\left[-K_{fdx}\dot{x}+u_{x}\left(t\right)u_{z}\left(t\right)\right]\;\ddot{y}\left(t\right)=\frac{1}{m}\left[-K_{fdy}\dot{y}+u_{y}\left(t\right)u_{z}\left(t\right)\right]\;\ddot{z}\left(t\right)=\frac{1}{m}\left[-K_{fdz}\dot{z}+\left(C_{\phi }C_{\theta }\right)u_{z}\left(t\right)\right]-g\;\ddot{\phi }\left(t\right)=\frac{1}{I_{x}}\left[\left(I_{y}-I_{z}\right)\dot{\psi }\dot{\theta }-K_{fax}{\dot{\phi }}^{2}-J_{r}\bar{\Omega }\dot{\theta }+du_{\phi }\left(t\right)\right]\;\ddot{\theta }\left(t\right)=\frac{1}{I_{y}}\left[\left(I_{z}-I_{x}\right)\dot{\psi }\dot{\phi }-K_{fay}{\dot{\theta }}^{2}+J_{r}\bar{\Omega }\dot{\phi }+du_{\theta }\left(t\right)\right]\;\ddot{\psi }\left(t\right)=\frac{1}{I_{z}}\left[\left(I_{x}-I_{y}\right)\dot{\phi }\dot{\theta }-K_{faz}{\dot{\psi }}^{2}+C_{D}u_{\psi }\left(t\right)\right]$$

where$\;S_{\phi }$ = sinϕ, $C_{\phi }$ = cosϕ, $S_{\theta }$ = sinθ, $C_{\theta }$ = cosθ, $S_{\psi }$ = sinψ,$\;C_{\psi }=cos\psi ,$ and $\bar{\Omega }=w_{1}-w_{2}+w_{3}-w_{4}$. The terms of $u_{z}$, $u_{\phi }$, $u_{\theta },$ and $u_{\psi }$ signify the control inputs of the quadrotor and $u_{x}\left(t\right)$ and $u_{y}\left(t\right)$ are the supplementary control inputs. The parameters of the dynamical model of quadrotor are given in Table 1.

In the quadrotor system, the following relations hold between angular velocities and control inputs:

(3) $$u_{z}\left(t\right)=K_{p}\left(w_{1}^{2}+w_{2}^{2}+w_{3}^{2}+w_{4}^{2}\right),\;u_{\phi }\left(t\right)=-K_{p}w_{1}^{2}+K_{p}w_{3}^{2},\;u_{\theta }\left(t\right)=-K_{p}w_{2}^{2}+K_{p}w_{4}^{2},\;u_{\psi }\left(t\right)=C_{d}\left(w_{1}^{2}-w_{2}^{2}+w_{3}^{2}-w_{4}^{2}\right)$$

By definition of the new variables as $\alpha_{1}=\frac{-K_{fdx}}{m}$, $\alpha_{2}=\frac{-K_{fdy}}{m}$, $\alpha_{3}=\frac{-K_{fdz}}{m}$,$\;\alpha_{4}=\frac{I_{y}-I_{z}}{I_{x}}$,$\;\alpha_{5}=\frac{-K_{fax}}{I_{x}}$,$\;\alpha_{6}=-\frac{J_{r}}{I_{x}}$, $\alpha_{7}=\frac{I_{z}-I_{x}}{I_{y}}$,$\;\alpha_{8}=\frac{-K_{fay}}{I_{y}}$,$\;\alpha_{9}=\frac{J_{r}}{I_{y}}\;,\;\alpha_{10}=\frac{I_{x}-I_{y}}{I_{z}}$, $\alpha_{11}=\frac{-K_{faz}}{I_{z}}$, $\beta_{1}=\frac{d}{I_{x}}$, $\beta_{2}=\frac{d}{I_{y}}$, and $\beta_{3}=\frac{C_{D}}{I_{z}}$, the dynamical equations are rewritten as:

(4) $$\ddot{x}\left(t\right)=\alpha_{1}\dot{x}\left(t\right)+\frac{u_{z}}{m}u_{x}\left(t\right),\;\ddot{y}\left(t\right)=\alpha_{2}\dot{y}\left(t\right)+\frac{u_{z}}{m}u_{y}\left(t\right),\;\ddot{z}\left(t\right)=\alpha_{3}\dot{z}\left(t\right)-g+\frac{\left(C_{\phi }C_{\theta }\right)}{m}u_{z}\left(t\right),\;\ddot{\phi }\left(t\right)=\alpha_{4}\dot{\psi }\left(t\right)\dot{\theta }\left(t\right)+\alpha_{5}{\dot{\phi }}^{2}\left(t\right)+\alpha_{6}\bar{\Omega }\dot{\theta }\left(t\right)+\beta_{1}u_{\phi }\left(t\right),\;\ddot{\theta }\left(t\right)=\alpha_{7}\dot{\psi }\left(t\right)\dot{\phi }\left(t\right)+\alpha_{8}{\dot{\theta }}^{2}\left(t\right)+\alpha_{9}\bar{\Omega }\dot{\phi }\left(t\right)+\beta_{2}u_{\theta }\left(t\right),\;\ddot{\psi }\left(t\right)=\alpha_{10}\dot{\phi }\left(t\right)\dot{\theta }\left(t\right)+\alpha_{11}{\dot{\psi }}^{2}\left(t\right)+\beta_{3}u_{\psi }\left(t\right).$$

Now, consider the state-space vector of the quadrotor system as ${\left[x_{1},x_{2},x_{3},x_{4},x_{5},x_{6},x_{7},x_{8},x_{9},x_{10},x_{11},x_{12}\right]}^{T}=X\left(t\right)={\left[x,\dot{x},y,\dot{y},z,\dot{z},\phi ,\dot{\phi },\theta ,\dot{\theta },\psi ,\dot{\psi }\right]}^{T}$ and the vector of the wind perturbation as $D\left(t\right)={\left[d_{x},d_{y},d_{z},d_{\phi },d_{\theta },d_{\psi }\right]}^{T}$. Then, Equation (4) can be rewritten in the state-space form as

(5) $${\dot{x}}_{1}\left(t\right)=x_{2}\left(t\right),\;{\dot{x}}_{2}\left(t\right)=\alpha_{1}x_{2}\left(t\right)+\frac{u_{z}\left(t\right)}{m}u_{x}\left(t\right)+d_{x}\left(t\right),\;{\dot{x}}_{3}\left(t\right)=x_{4}\left(t\right),\;{\dot{x}}_{4}\left(t\right)=\alpha_{2}x_{4}\left(t\right)+\frac{u_{z}\left(t\right)}{m}u_{y}\left(t\right)+d_{y}\left(t\right),\;{\dot{x}}_{5}\left(t\right)=x_{6}\left(t\right),\;\;{\dot{x}}_{6}\left(t\right)=\alpha_{3}x_{6}\left(t\right)-g+\frac{C_{\phi }C_{\theta }}{m}u_{z}\left(t\right)+d_{z}\left(t\right),\;{\dot{x}}_{7}\left(t\right)=x_{8}\left(t\right),\;{\dot{x}}_{8}\left(t\right)=\alpha_{4}x_{12}\left(t\right)x_{10}\left(t\right)+\alpha_{5}x_{8}{}^{2}\left(t\right)+\alpha_{6}\bar{\Omega }x_{10}\left(t\right)+\beta_{1}u_{\phi }\left(t\right)+d_{\phi }\left(t\right),\;{\dot{x}}_{9}\left(t\right)=x_{10}\left(t\right),\;{\dot{x}}_{10}\left(t\right)=\alpha_{7}x_{12}\left(t\right)x_{8}\left(t\right)+\alpha_{8}x_{10}{}^{2}\left(t\right)+\alpha_{9}\bar{\Omega }x_{8}\left(t\right)+\beta_{2}u_{\theta }\left(t\right)+d_{\theta }\left(t\right),\;{\dot{x}}_{11}\left(t\right)=x_{12}\left(t\right),\;{\dot{x}}_{12}\left(t\right)=\alpha_{10}x_{8}\left(t\right)x_{10}\left(t\right)+\alpha_{11}x_{12}{}^{2}\left(t\right)+\beta_{3}u_{\psi }\left(t\right)+d_{\psi }\left(t\right)$$

## 3. Disturbance Observer Design

In this part, for the rejection of the external disturbance related to the wind perturbation, a new disturbance observer was designed to estimate the perturbation at any moment. For this reason, the disturbance observer variable was defined as:

(6) $$V_{x}\left(t\right)=W_{x}\left(t\right)-x_{2}\left(t\right)\;V_{y}\left(t\right)=W_{y}\left(t\right)-x_{4}\left(t\right),\;V_{z}\left(t\right)=W_{z}\left(t\right)-x_{6}\left(t\right),\;V_{\phi }\left(t\right)=W_{\phi }\left(t\right)-x_{8}\left(t\right),\;V_{\theta }\left(t\right)=W_{\theta }\left(t\right)-x_{10}\left(t\right),\;V_{\psi }\left(t\right)=W_{\psi }\left(t\right)-x_{12}\left(t\right)$$

where $W_{i}\left(t\right)\;$is determined by the following law:

(7) W˙xt=−kxVxt−bxtsign(Vxt)−εxVxnxmxt+α1x2t+uztmuxtW˙yt=−kyVyt−bytsign(Vyt)−εyVynymyt+α2x4t+uztmuytW˙zt=−kzVzt−bztsign(Vzt)−εzVznzmzt+α3x6t−g+CϕCθmuztW˙ϕt=−kϕVϕt−bϕtsign(Vϕt)−εϕVϕnϕmϕt+α4x12tx10t+α5x82t+α6Ω¯x10t+β1uϕtW˙θt=−kθVθt−bθtsign(Vθt)−εθVθnθmθt+α7x12tx8t+α8x102t+α9Ω¯x8t+β2uθtW˙ψt=−kψVψt−bψtsign(Vψt)−εψVψnψmψt+α10x8tx10t+α11x122t+β3uψt,

where $n_{i}\prime s$ and $m_{i}\prime s$ are two odd positive numbers ($n_{i}<m_{i}$). The terms ${\dot{W}}_{i}\left(t\right)$ are defined as (7), where the time derivates of the disturbance observer variables $V_{i}\left(t\right)$ satisfy the finite time convergence criterion. The design coefficients $k_{i}\prime s$ and $\varepsilon_{i}\prime s$ are some positive constants. The disturbance observer ${\hat{d}}_{i}\left(t\right),\;\left(\forall i=x,y,z,\phi ,\theta ,\psi \right)$ is found as

(8) $${\hat{d}}_{i}\left(t\right)=-k_{i}V_{i}\left(t\right)-b_{i}\left(t\right)sign(V_{i}\left(t\right))-\varepsilon_{i}V_{i}{\left(t\right)}^{\frac{n_{i}}{m_{i}}}$$

where $b_{i}\geq {\left|d_{i}\right|}_{max}\geq \left|d_{i}\right|$.

**Theorem** **1.** *Consider the disturbed nonlinear quadrotor system (5) and the disturbance observer (8). Hence, the exterior disturbance related to the wind perturbation is estimated and the disturbance estimation error converges to zero in the finite time.*

**Proof.**  Taking the time derivative of (6), it yields:

(9) $${\dot{V}}_{x}\left(t\right)={\dot{W}}_{x}\left(t\right)-{\dot{x}}_{2}\left(t\right)\;{\dot{V}}_{y}\left(t\right)={\dot{W}}_{y}\left(t\right)-{\dot{x}}_{4}\left(t\right),\;{\dot{V}}_{z}\left(t\right)={\dot{W}}_{z}\left(t\right)-{\dot{x}}_{6}\left(t\right),\;{\dot{V}}_{\phi }\left(t\right)={\dot{W}}_{\phi }\left(t\right)-{\dot{x}}_{8}\left(t\right),\;{\dot{V}}_{\theta }\left(t\right)={\dot{W}}_{\theta }\left(t\right)-{\dot{x}}_{10}\left(t\right),\;{\dot{V}}_{\psi }\left(t\right)={\dot{W}}_{\psi }\left(t\right)-{\dot{x}}_{12}\left(t\right)$$

where using (5) and (7), we have:

(10) V˙xt=−kxVxt−bxtsign(Vxt)−εxVxnxmxt+α1x2t+uztmuxt−α1x2t−uztmuxt−dxtV˙yt=−kyVyt−bytsign(Vyt)−εyVynymyt+α2x4t+uztmuyt−α2x4t−uztmuyt−dytV˙zt=−kzVzt−bztsign(Vzt)−εzVznzmzt+α3x6t−g+CϕCθmuzt−α3x6t+g−CϕCθmuzt−dztV˙ϕt=[−kϕVϕt−bϕtsign(Vϕt)−εϕVϕnϕmϕt+α4x12tx10t+α5x82t+α6Ω¯x10t+β1uϕt−α4x12tx10t−α5x82t−α6Ω¯x10t−β1uϕt−dϕt]V˙θt=[−kθVθt−bθtsign(Vθt)−εθVθnθmθt+α7x12tx8t+α8x102t+α9Ω¯x8t+β2uθt−α7x12tx8t−α8x102t−α9Ω¯x8t−β2uθt−dθt]V˙ψt=−kψVψt−bψtsign(Vψt)−εψVψnψmψt+α10x8tx10t+α11x122t+β3uψt−α10x8tx10t−α11x122t−β3uψt−dψt

Removing the similar expression leads to:

(11) $${\dot{V}}_{i}\left(t\right)=-k_{i}V_{i}\left(t\right)-b_{i}\left(t\right)sign(V_{i}\left(t\right))-\varepsilon_{i}V_{i}{\left(t\right)}^{\frac{n_{i}}{m_{i}}}-d_{i}\left(t\right).$$

Considering the positive-definite Lyapunov function as:

(12) $$L_{1}{}_{i}\left(V_{i}\left(t\right)\right)=0.5V_{i}{\left(t\right)}^{2}$$

where taking time derivative of (12) and using (11), the following result is found as:

(13) $${\dot{L}}_{1}{}_{i}\left(V_{i}\left(t\right)\right)=V_{i}\left(t\right)\left(-k_{i}V_{i}\left(t\right)-b_{i}\left(t\right)sign(V_{i}\left(t\right)\right)-\varepsilon_{i}V_{i}{\left(t\right)}^{\frac{n_{i}}{m_{i}}}-d_{i}\left(t\right))$$

After simplification, it can obtain:

(14) $${\dot{L}}_{1}{}_{i}\left(V_{i}\left(t\right)\right)\leq -k_{i}V_{i}{}^{2}\left(t\right)-b_{i}\left(t\right)\left|V_{i}\left(t\right)\right|-\varepsilon_{i}V_{i}{\left(t\right)}^{\frac{n_{i}}{m_{i}}+1}-d_{i}\left(t\right)\left|V_{i}\left(t\right)\right|$$

whereas $-d_{i}\left(t\right)\left|V_{i}\left(t\right)\right|\leq \left|d_{i}\left(t\right)\right|\left|V_{i}\left(t\right)\right|$, so it can gain:

(15) $${\dot{L}}_{1}{}_{i}\left(V_{i}\left(t\right)\right)\leq -k_{i}V_{i}{}^{2}\left(t\right)-b_{i}\left(t\right)\left|V_{i}\left(t\right)\right|-\varepsilon_{i}V_{i}{\left(t\right)}^{\frac{n_{i}}{m_{i}}+1}+\left|d_{i}\left(t\right)\right|\left|V_{i}\left(t\right)\right|$$

Based on the assumption $b_{i}\geq {\left|d_{i}\right|}_{max}\geq \left|d_{i}\right|$, we have:

(16) $${\dot{L}}_{1}{}_{i}\left(V_{i}\left(t\right)\right)\leq -k_{i}V_{i}{}^{2}\left(t\right)-b_{i}\left(t\right)\left|V_{i}\left(t\right)\right|-\varepsilon_{i}V_{i}{\left(t\right)}^{\frac{n_{i}}{m_{i}}+1}+b_{i}\left(t\right)\left|V_{i}\left(t\right)\right|$$

where removing the same terms of the above equation leads to:

(17) $${\dot{L}}_{1}{}_{i}\left(V_{i}\left(t\right)\right)\leq -k_{i}V_{i}{}^{2}\left(t\right)-\varepsilon_{i}V_{i}{\left(t\right)}^{\frac{n_{i}}{m_{i}}+1}$$

According to Equation (17), it can be concluded that the disturbance observer variable $V_{i}\left(t\right)$ converges to zero in the finite time. The disturbance estimation error can be calculated by:

(18) $${\tilde{d}}_{i}\left(t\right)={\hat{d}}_{i}\left(t\right)-d_{i}\left(t\right)$$

Using (5) and (8) and substituting them into (18), one can gain:

(19) d˜xt=−kxVxt−bxtsign(Vxt)−εxVxnxmxt+α1x2t+uztmuxt−x˙2td˜yt=−kyVyt−bytsign(Vyt)−εyVynymyt+α2x4t+uztmuyt−x˙4td˜zt=−kzVzt−bztsign(Vzt)−εzVznzmzt+α3x6t−g+CϕCθmuzt−x˙6td˜ϕt=−kϕVϕt−bϕtsign(Vϕt)−εϕVϕnϕmϕt+α4x12tx10t+α5x82t+α6Ω¯x10t+β1uϕt−x˙8td˜θt=−kθVθt−bθtsign(Vθt)−εθVθnθmθt+α7x12tx8t+α8x102t+α9Ω¯x8t+β2uθt−x˙10td˜ψt=−kψVψt−bψtsign(Vψt)−εψVψnψmψt+α10x8tx10t+α11x122t+β3uψt−x˙12t

Considering the Equation (7), it attains:

(20) $${\tilde{d}}_{x}\left(t\right)={\dot{W}}_{x}\left(t\right)-{\dot{x}}_{2}\left(t\right),\;{\tilde{d}}_{y}\left(t\right)={\dot{W}}_{y}\left(t\right)-{\dot{x}}_{4}\left(t\right),\;{\tilde{d}}_{z}\left(t\right)={\dot{W}}_{z}\left(t\right)-{\dot{x}}_{6}\left(t\right),\;{\tilde{d}}_{\phi }\left(t\right)={\dot{W}}_{\phi }\left(t\right)-{\dot{x}}_{8}\left(t\right),\;{\tilde{d}}_{\theta }\left(t\right)={\dot{W}}_{\theta }\left(t\right)-{\dot{x}}_{10}\left(t\right),\;{\tilde{d}}_{\psi }\left(t\right)={\dot{W}}_{\psi }\left(t\right)-{\dot{x}}_{12}\left(t\right)$$

Respect to the Equation (9), we obtain:

(21) $${\tilde{d}}_{i}\left(t\right)={\dot{V}}_{i}\left(t\right)$$

Since the disturbance observer variable $V_{i}\left(t\right)$ converges to origin in the finite time (Equation (17)), then the time derivative of $V_{i}\left(t\right)$ becomes zero in the finite time, i.e., ${\dot{V}}_{i}\left(t\right)=0$, and the estimation error ${\tilde{d}}_{i}\left(t\right)$ reaches zero. Therefore, the disturbance observer (8) estimates the exterior disturbance related to the wind perturbation. □

## 4. Non-singular Terminal Sliding Mode Control

In this paper, the main control objective was the tracking control of the quadrotor in the presence of wind perturbation based on the non-singular terminal SMC using disturbance observer. For this reason, tracking error was defined as

(22) $$E_{x}\left(t\right)=x_{1}\left(t\right)-x_{d}\left(t\right),\;E_{y}\left(t\right)=x_{3}\left(t\right)-y_{d}\left(t\right),\;E_{z}\left(t\right)=x_{5}\left(t\right)-z_{d}\left(t\right),\;E_{\phi }\left(t\right)=x_{7}\left(t\right)-\phi_{d}\left(t\right),\;E_{\theta }\left(t\right)=x_{9}\left(t\right)-\theta_{d}\left(t\right),\;E_{\psi }\left(t\right)=x_{11}\left(t\right)-\psi_{d}\left(t\right)$$

where $x_{d}\left(t\right)$, $y_{d}\left(t\right)$, $z_{d}\left(t\right)$, $\phi_{d}\left(t\right)$, $\theta_{d}\left(t\right),$ and $\psi_{d}\left(t\right)$ are desired values, and the non-singular terminal sliding mode surface is defined as:

(23) $$\sigma_{i}\left(t\right)={\dot{E}}_{i}\left(t\right)+\ell_{i}E_{i}\left(t\right)+\gamma_{i}\int_{0}^{t}\left(E_{i}{}^{\eta_{1}}\left(\tau \right)+{\dot{E}}_{i}{}^{\eta_{2}}\left(\tau \right)\right)d\tau +V_{i}\left(t\right)$$

with $\ell_{i}$ and $\gamma_{i}$ as the positive constants $\left(\forall i=x,y,z,\;\phi ,\theta ,\psi \right)$, and$\;\eta_{1}=\frac{c_{1}}{c_{2}}$,$\eta_{2}=\frac{c_{3}}{c_{4}}$ where $c_{i}$’s are the odd integer positive constants with $c_{1}<c_{2}$ and $c_{3}<c_{4}$.

In order to satisfy the finite time convergence of non-singular terminal sliding surface (23) to the origin, the subsequent theorem is provided:

**Theorem** **2.** *Consider the position and attitude dynamical model of the quadrotor under wind perturbation as (5) and the non-singular terminal sliding surface (23). If the finite time position and attitude controllers with the rapid reaching law are designed as:*

(24) $$u_{x}\left(t\right)=-\frac{m}{u_{z}\left(t\right)}\left[\alpha_{1}x_{2}\left(t\right)-{\ddot{x}}_{d}\left(t\right)+{\hat{d}}_{x}\left(t\right)+\ell_{x}{\dot{E}}_{x}\left(t\right)+\gamma_{x}\left(E_{x}{}^{\eta_{1}}\left(t\right)+{\dot{E}}_{x}{}^{\eta_{2}}\left(t\right)\right)\;+b_{1x}\left(C_{x}{}^{\left|\sigma_{x}\left(t\right)\right|}-1\right)sign\left(\sigma_{x}\left(t\right)\right)+b_{2x}{\left|\sigma_{x}\left(t\right)\right|}^{a_{x}}sign\left(\sigma_{x}\left(t\right)\right)\right]\;u_{y}\left(t\right)=-\frac{m}{u_{z}\left(t\right)}\left[\alpha_{2}x_{4}\left(t\right)-{\ddot{y}}_{d}\left(t\right)+{\hat{d}}_{y}\left(t\right)+\ell_{y}{\dot{E}}_{y}\left(t\right)+\gamma_{y}\left(E_{y}{}^{\eta_{1}}\left(t\right)+{\dot{E}}_{y}{}^{\eta_{2}}\left(t\right)\right)\;+b_{1y}\left(C_{y}{}^{\left|\sigma_{y}\left(t\right)\right|}-1\right)sign\left(\sigma_{y}\left(t\right)\right)+b_{2y}{\left|\sigma_{y}\left(t\right)\right|}^{a_{y}}sign\left(\sigma_{y}\left(t\right)\right)\right]\;u_{z}\left(t\right)=-\frac{m}{C_{\phi }C_{\theta }}\left[\alpha_{3}x_{6}\left(t\right)-g-{\ddot{z}}_{d}\left(t\right)+{\hat{d}}_{z}\left(t\right)+\ell_{z}{\dot{E}}_{z}\left(t\right)+\gamma_{z}\left(E_{z}{}^{\eta_{1}}\left(t\right)+{\dot{E}}_{z}{}^{\eta_{2}}\left(t\right)\right)\;+b_{1z}\left(C_{z}{}^{\left|\sigma_{z}\left(t\right)\right|}-1\right)sign\left(\sigma_{z}\left(t\right)\right)+b_{2z}{\left|\sigma_{z}\left(t\right)\right|}^{a_{z}}sign\left(\sigma_{z}\left(t\right)\right)\right]\;\begin{array}{ll} u_{\phi }\left(t\right)=-\frac{1}{\beta_{1}}[\alpha_{4} & x_{12}\left(t\right)x_{10}\left(t\right)+\alpha_{5}x_{8}{}^{2}\left(t\right)+\alpha_{6}\bar{\Omega }x_{10}\left(t\right)-{\ddot{\phi }}_{d}\left(t\right)\\ & +{\hat{d}}_{\phi }\left(t\right)+\ell_{\phi }{\dot{E}}_{\phi }\left(t\right)+\gamma_{\phi }\left(E_{\phi }{}^{\eta_{1}}\left(t\right)+{\dot{E}}_{\phi }{}^{\eta_{2}}\left(t\right)\right)\\ & +b_{1\phi }\left(C_{\phi }{}^{\left|\sigma_{\phi }\left(t\right)\right|}-1\right)sign\left(\sigma_{\phi }\left(t\right)\right)\\ & +b_{2\phi }{\left|\sigma_{\phi }\left(t\right)\right|}^{a_{\phi }}sign\left(\sigma_{\phi }\left(t\right)\right)] \end{array}\;\begin{array}{ll} u_{\theta }\left(t\right)=-\frac{1}{\beta_{2}}[\alpha_{7} & x_{12}\left(t\right)x_{8}\left(t\right)+\alpha_{8}x_{10}{}^{2}\left(t\right)+\alpha_{9}\bar{\Omega }x_{8}\left(t\right)-{\ddot{\theta }}_{d}\left(t\right)+{\hat{d}}_{\theta }\left(t\right)+\ell_{\theta }{\dot{E}}_{\theta }\left(t\right)\\ & +\gamma_{\theta }\left(E_{\theta }{}^{\eta_{1}}\left(t\right)+{\dot{E}}_{\theta }{}^{\eta_{2}}\left(t\right)\right)+b_{1\theta }\left(C_{\theta }{}^{\left|\sigma_{\theta }\left(t\right)\right|}-1\right)sign\left(\sigma_{\theta }\left(t\right)\right)\\ & +b_{2\theta }{\left|\sigma_{\theta }\left(t\right)\right|}^{a_{\theta }}sign\left(\sigma_{\theta }\left(t\right)\right)] \end{array}\;\begin{array}{ll} u_{\psi }\left(t\right)=-\frac{1}{\beta_{3}}[\alpha_{10} & x_{8}\left(t\right)x_{10}\left(t\right)+\alpha_{11}x_{12}{}^{2}\left(t\right)-{\ddot{\psi }}_{d}\left(t\right)+{\hat{d}}_{\psi }\left(t\right)+\ell_{\psi }{\dot{E}}_{\psi }\left(t\right)\\ & +\gamma_{\psi }\left(E_{\psi }{}^{\eta_{1}}\left(t\right)+{\dot{E}}_{\psi }{}^{\eta_{2}}\left(t\right)\right)+b_{1\psi }\left(C_{\psi }{}^{\left|\sigma_{\psi }\left(t\right)\right|}-1\right)sign\left(\sigma_{\psi }\left(t\right)\right)\\ & +b_{2\psi }{\left|\sigma_{\psi }\left(t\right)\right|}^{a_{\psi }}sign\left(\sigma_{\psi }\left(t\right)\right)] \end{array}$$

*with*$b_{1i},b_{2i}>0$, $0<a_{i}<1$*and*$C_{i}=1+\left(b_{2i}/b_{1i}\right)$*, then the non-singular TSMC surface reaches zero in the finite time and the reachability condition is satisfied.*

**Proof.**  Time derivative of non-singular terminal sliding surfaces (23) is obtained as:

(25) $${\dot{\sigma }}_{i}\left(t\right)={\ddot{E}}_{i}\left(t\right)+\ell_{i}{\dot{E}}_{i}\left(t\right)+\gamma_{i}\left(E_{i}{}^{\eta_{1}}\left(\tau \right)+{\dot{E}}_{i}{}^{\eta_{2}}\left(\tau \right)\right)+{\dot{V}}_{i}\left(t\right)$$

where applying (5) and (22), we have

(26) $${\dot{\sigma }}_{x}\left(t\right)=\left[\alpha_{1}x_{2}\left(t\right)+\frac{u_{z}\left(t\right)}{m}u_{x}\left(t\right)-{\ddot{x}}_{d}\left(t\right)+\ell_{x}{\dot{E}}_{x}\left(t\right)+\gamma_{x}\left(E_{x}{}^{\eta_{1}}\left(t\right)+{\dot{E}}_{x}{}^{\eta_{2}}\left(t\right)\right)\;+{\dot{V}}_{x}\left(t\right)+d_{x}\right]\;{\dot{\sigma }}_{y}\left(t\right)=\left[\alpha_{2}x_{4}\left(t\right)+\frac{u_{z}\left(t\right)}{m}u_{y}\left(t\right)-{\ddot{y}}_{d}\left(t\right)+\ell_{y}{\dot{E}}_{y}\left(t\right)+\gamma_{y}\left(E_{y}{}^{\eta_{1}}\left(t\right)+{\dot{E}}_{y}{}^{\eta_{2}}\left(t\right)\right)\;+{\dot{V}}_{y}\left(t\right)+d_{y}\right]\;{\dot{\sigma }}_{z}\left(t\right)=\left[\alpha_{3}x_{6}\left(t\right)-g+\frac{\left(C_{\phi }C_{\theta }\right)}{m}u_{z}\left(t\right)-{\ddot{z}}_{d}\left(t\right)+\ell_{z}{\dot{E}}_{z}\left(t\right)+\gamma_{z}(E_{z}{}^{\eta_{1}}\left(t\right)\;+{\dot{E}}_{z}{}^{\eta_{2}}\left(t\right))+{\dot{V}}_{z}\left(t\right)+d_{z}\right]\;{\dot{\sigma }}_{\phi }\left(t\right)=\left[\alpha_{4}x_{12}\left(t\right)x_{10}\left(t\right)+\alpha_{5}x_{8}{}^{2}\left(t\right)+\alpha_{6}\bar{\Omega }x_{10}\left(t\right)+\beta_{1}u_{\phi }\left(t\right)-{\ddot{\phi }}_{d}\left(t\right)+\ell_{\phi }{\dot{E}}_{\phi }\left(t\right)\;+\gamma_{\phi }\left(E_{\phi }{}^{\eta_{1}}\left(t\right)+{\dot{E}}_{\phi }{}^{\eta_{2}}\left(t\right)\right)+{\dot{V}}_{\phi }\left(t\right)+d_{\phi }\right]\;{\dot{\sigma }}_{\theta }\left(t\right)=\left[\alpha_{7}x_{12}\left(t\right)x_{8}\left(t\right)+\alpha_{8}x_{10}{}^{2}\left(t\right)+\alpha_{9}\bar{\Omega }x_{8}\left(t\right)+\beta_{2}u_{\theta }\left(t\right)-{\ddot{\theta }}_{d}\left(t\right)+\ell_{\theta }{\dot{E}}_{\theta }\left(t\right)\;+\gamma_{\theta }\left(E_{\theta }{}^{\eta_{1}}\left(t\right)+{\dot{E}}_{\theta }{}^{\eta_{2}}\left(t\right)\right)+{\dot{V}}_{\theta }\left(t\right)+d_{\theta }\right]\;{\dot{\sigma }}_{\psi }\left(t\right)=\left[\alpha_{10}x_{8}\left(t\right)x_{10}\left(t\right)+\alpha_{11}x_{12}{}^{2}\left(t\right)+\beta_{3}u_{\psi }\left(t\right)-{\ddot{\psi }}_{d}\left(t\right)+\ell_{\psi }{\dot{E}}_{\psi }\left(t\right)\;+\gamma_{\psi }\left(E_{\psi }{}^{\eta_{1}}\left(t\right)+{\dot{E}}_{\psi }{}^{\eta_{2}}\left(t\right)\right)+{\dot{V}}_{\psi }\left(t\right)+d_{\psi }\right]$$

From (21) one attains:

(27) $${\hat{d}}_{i}\left(t\right)=d_{i}\left(t\right)+{\dot{V}}_{i}\left(t\right)$$

Substitution of (27) into (26), it can achieve:

(28) $${\dot{\sigma }}_{x}\left(t\right)=\left[\alpha_{1}x_{2}\left(t\right)+\frac{u_{z}\left(t\right)}{m}u_{x}\left(t\right)-{\ddot{x}}_{d}\left(t\right)+\ell_{x}{\dot{E}}_{x}\left(t\right)+\gamma_{x}\left(E_{x}{}^{\eta_{1}}\left(t\right)+{\dot{E}}_{x}{}^{\eta_{2}}\left(t\right)\right)+{\hat{d}}_{x}\left(t\right)\right]\;{\dot{\sigma }}_{y}\left(t\right)=\left[\alpha_{2}x_{4}\left(t\right)+\frac{u_{z}\left(t\right)}{m}u_{y}\left(t\right)-{\ddot{y}}_{d}\left(t\right)+\ell_{y}{\dot{E}}_{y}\left(t\right)+\gamma_{y}\left(E_{y}{}^{\eta_{1}}\left(t\right)+{\dot{E}}_{y}{}^{\eta_{2}}\left(t\right)\right)+{\hat{d}}_{y}\left(t\right)\right]\;{\dot{\sigma }}_{z}\left(t\right)=\left[\alpha_{3}x_{6}\left(t\right)-g+\frac{\left(C_{\phi }C_{\theta }\right)}{m}u_{z}\left(t\right)-{\ddot{z}}_{d}\left(t\right)+\ell_{z}{\dot{E}}_{z}\left(t\right)+\gamma_{z}\left(E_{z}{}^{\eta_{1}}\left(t\right)\;+{\dot{E}}_{z}{}^{\eta_{2}}\left(t\right)\right)+{\hat{d}}_{z}\left(t\right)\right]\;{\dot{\sigma }}_{\phi }\left(t\right)=\left[\alpha_{4}x_{12}\left(t\right)x_{10}\left(t\right)+\alpha_{5}x_{8}{}^{2}\left(t\right)+\alpha_{6}\bar{\Omega }x_{10}\left(t\right)+\beta_{1}u_{\phi }\left(t\right)-{\ddot{\phi }}_{d}\left(t\right)\;+\ell_{\phi }{\dot{E}}_{\phi }\left(t\right)+\gamma_{\phi }\left(E_{\phi }{}^{\eta_{1}}\left(t\right)+{\dot{E}}_{\phi }{}^{\eta_{2}}\left(t\right)\right)+{\hat{d}}_{\phi }\left(t\right)\right]\;{\dot{\sigma }}_{\theta }\left(t\right)=\left[\alpha_{7}x_{12}\left(t\right)x_{8}\left(t\right)+\alpha_{8}x_{10}{}^{2}\left(t\right)+\alpha_{9}\bar{\Omega }x_{8}\left(t\right)+\beta_{2}u_{\theta }\left(t\right)-{\ddot{\theta }}_{d}\left(t\right)\;+\ell_{\theta }{\dot{E}}_{\theta }\left(t\right)+\gamma_{\theta }\left(E_{\theta }{}^{\eta_{1}}\left(t\right)+{\dot{E}}_{\theta }{}^{\eta_{2}}\left(t\right)\right)+{\hat{d}}_{\theta }\left(t\right)\right]\;{\dot{\sigma }}_{\psi }\left(t\right)=\left[\alpha_{10}x_{8}\left(t\right)x_{10}\left(t\right)+\alpha_{11}x_{12}{}^{2}\left(t\right)+\beta_{3}u_{\psi }\left(t\right)-{\ddot{\psi }}_{d}\left(t\right)+\ell_{\psi }{\dot{E}}_{\psi }\left(t\right)+\;\gamma_{\psi }\left(E_{\psi }{}^{\eta_{1}}\left(t\right)+{\dot{E}}_{\psi }{}^{\eta_{2}}\left(t\right)\right)+{\hat{d}}_{\psi }\left(t\right)\right].$$

Substituting the non-singular terminal sliding mode controller (24) in (28), one can find:

(29) $${\dot{\sigma }}_{i}\left(t\right)=-b_{1i}\left(C_{i}{}^{\left|\sigma_{i}\left(t\right)\right|}-1\right)sign(\sigma_{i}\left(t\right))-b_{2i}{\left|\sigma_{i}\left(t\right)\right|}^{a_{i}}sign(\sigma_{i}\left(t\right))$$

Construct the Lyapunov function as:

(30) $$L_{3}{}_{i}\left(t\right)=0.5\sigma_{i}{}^{2}\left(t\right)$$

where differentiating (30) and using (29) give:

(31) $${\dot{L}}_{3}{}_{i}\left(t\right)=\sigma_{i}\left(t\right)\left(-b_{1i}\left(C_{i}{}^{\left|\sigma_{i}\left(t\right)\right|}-1\right)sign(\sigma_{i}\left(t\right)\right)-b_{2i}{\left|\sigma_{i}\left(t\right)\right|}^{a_{i}}sign(\sigma_{i}\left(t\right))),$$

which leads to:

(32) $${\dot{L}}_{3}{}_{i}\left(t\right)=-b_{1i}\left(C_{i}{}^{\left|\sigma_{i}\left(t\right)\right|}-1\right)\left|\sigma_{i}\left(t\right)\right|-b_{2i}{\left|\sigma_{i}\left(t\right)\right|}^{a_{i}+1}$$

From (30) the term $\left|\sigma_{i}\left(t\right)\right|$ is equal to $2^{0.5}L_{3}{{}_{i}}^{0.5}\left(t\right)$. Therefore, Equation (32) is written as:

(33) $${\dot{L}}_{3}{}_{i}\left(t\right)=-2^{0.5}b_{1i}\left(C_{i}{}^{\left|\sigma_{i}\left(t\right)\right|}-1\right)L_{3}{{}_{i}}^{0.5}\left(t\right)-2^{0.5\left(a_{i}+1\right)}b_{2i}L_{3}{{}_{i}}^{0.5\left(a_{i}+1\right)}\left(t\right)<0$$

where it guarantees that the non-singular terminal sliding (23) with the fast reaching law is convergent to the origin in the finite time. □

In the non-singular terminal sliding mode stabilizing controllers (24), two significant terms are given, i.e., $b_{1i}\left(C_{i}{}^{\left|\sigma_{i}\left(t\right)\right|}-1\right)sign(\sigma_{i}\left(t\right))$ and $b_{2i}{\left|\sigma_{i}\left(t\right)\right|}^{a_{i}}sign(\sigma_{i}\left(t\right))$. Using these terms, the rapid reaching law is found as:

(34) $${\dot{\sigma }}_{i}\left(t\right)=-b_{1i}\left(C_{i}{}^{\left|\sigma_{i}\left(t\right)\right|}-1\right)sign(\sigma_{i}\left(t\right))-b_{2i}{\left|\sigma_{i}\left(t\right)\right|}^{a_{i}}sign(\sigma_{i}\left(t\right))$$

When $\left|\sigma_{i}\left(t\right)\right|>1$, the first sentence in (34) becomes the dominant law and the change rate of the first term is larger than that of the second term; then, it speeds up the reaching rate. When $\left|\sigma_{i}\left(t\right)\right|<1$, the second sentence in (34) plays a dominant role and increases the accuracy rate. When the initial value of the non-singular terminal sliding surface is greater than one, that is, $\sigma_{i}\left(t_{0}\right)>1$, the motion process from the initial value to the sliding mode is separated to two phases as follows:

*Phase* (*a*): $\sigma_{i}\left(t_{0}\right)\to \sigma_{i}\left(t\right)=1$. One attains $\sigma_{i}\left(t\right)>1$; then $b_{1i}\left(C_{i}{}^{\left|\sigma_{i}\left(t\right)\right|}-1\right)>b_{2i}{\left|\sigma_{i}\left(t\right)\right|}^{a_{i}}$ is true and the second term of (34) is neglected. Then, the fast reaching law (34) is changed to:

(35) $${\dot{\sigma }}_{i}\left(t\right)\approx -b_{1i}\left(C_{i}{}^{\left|\sigma_{i}\left(t\right)\right|}-1\right)$$

where by integrating it, we have:

(36) $$\int_{0}^{t_{i}}dt\approx -\frac{1}{b_{1i}\ln C_{i}}\int_{\sigma_{i}\left(t_{0}\right)}^{1}d\left(ln\left(1-C_{i}{}^{-\sigma_{i}\left(\tau \right)}\right)\right)d\tau$$

Therefore, the convergence time of this phase is calculated by:

(37) $$t_{i}\approx \frac{ln\left(1-C_{i}{}^{-\sigma_{i}\left(t_{0}\right)}\right)-ln\left(1-C_{i}{}^{-1}\right)}{b_{1i}lnC_{i}}$$

*Phase* (*b*): $\sigma_{i}\left(t\right)=1\to \sigma_{i}\left(t\right)=0$. In this phase, we obtain $b_{1i}\left(C_{i}{}^{\left|\sigma_{i}\left(t\right)\right|}-1\right)<b_{2i}{\left|\sigma_{i}\left(t\right)\right|}^{a_{i}}$. Then, the second term in (34) has a prominent role. Thus, the reaching law (34) is changed to:

(38) $${\dot{\sigma }}_{i}\left(t\right)\approx -b_{2i}{\left|\sigma_{i}\left(t\right)\right|}^{a_{i}}$$

Taking integration of Equation (38), one achieves:

(39) $$\int_{0}^{t_{j}}dt\approx -\frac{1}{b_{2i}}\int_{1}^{0}\frac{1}{\sigma_{i}{\left(t\right)}^{a_{i}}}d\sigma_{i}\left(\tau \right)$$

The convergence time of this phase is calculated as:

(40) $$t_{j}\approx \frac{1}{b_{2i}\left(1-a_{i}\right)}$$

Therefore, the total convergence time $t_{T}$ is found as:

(41) $$t_{T}\approx t_{i}+t_{j}=\frac{ln\left(1-C_{i}{}^{-\sigma_{i}\left(t_{0}\right)}\right)-ln\left(1-C_{i}{}^{-1}\right)}{b_{1i}lnC_{i}}+\frac{1}{b_{2i}\left(1-a_{i}\right)}$$

Additionally, when the initial sliding surface is less than −1, namely $\sigma_{i}\left(t_{0}\right)<-1$, the motion process from the initial states to the sliding mode is separated to the following phases:

*Phase* (*a*’) $\sigma_{i}\left(t_{0}\right)\to \sigma_{i}\left(t\right)=-1$. We have $\sigma_{i}<-1$ and $b_{1i}\left(C_{i}{}^{\left|\sigma_{i}\left(t\right)\right|}-1\right)>b_{2i}{\left|\sigma_{i}\left(t\right)\right|}^{a_{i}}$; then, the first term of (34) has a dominant effect and the second term is ignored. Consequently, the fast reaching law (34) is written as:

(42) $${\dot{\sigma }}_{i}\left(t\right)\approx b_{1i}\left(C_{i}{}^{\left|\sigma_{i}\left(t\right)\right|}-1\right)$$

where by integrating (42), one has:

(43) $$\int_{0}^{t_{i\prime }}dt\approx -\frac{1}{b_{1i}lnC_{i}}\int_{\sigma_{i}\left(t_{0}\right)}^{-1}d\left(ln\left(1-C_{i}{}^{\sigma_{i}\left(t\right)}\right)\right)$$

The convergence time is then calculated as:

(44) $$t_{i\prime }\approx \frac{ln\left(1-C_{i}{}^{\sigma_{i}\left(t\right)}\right)-ln\left(1-C_{i}{}^{-1}\right)}{b_{1i}lnC_{i}}$$

*Phase* (*b’*) $\sigma_{i}\left(t\right)=-1\to \sigma_{i}\left(t\right)=0$. In this phase, one has $b_{1i}\left(C_{i}{}^{\left|\sigma_{i}\left(t\right)\right|}-1\right)<b_{2i}{\left|\sigma_{i}\left(t\right)\right|}^{a_{i}}$. Thus, the second term of (34) has the main role. The fast reaching law (34) is changed to:

(45) $${\dot{\sigma }}_{i}\left(t\right)\approx b_{2i}{\left(-\sigma_{i}\left(t\right)\right)}^{a_{i}}$$

where taking integral of the above equation, we have:

(46) $$\int_{0}^{t_{j\prime }}dt\approx \frac{1}{b_{2i}}\int_{-1}^{0}\frac{1}{{\left(-\sigma_{i}\left(\tau \right)\right)}^{a_{i}}}d\sigma \left(\tau \right)$$

The convergence time of this phase is calculated as:

(47) $$t_{j\prime }\approx \frac{1}{b_{2i}\left(1-a_{i}\right)}$$

Thus, the resulted convergence time is calculated by:

(48) $$t_{T\prime }\approx t_{i\prime }+t_{j\prime }=\frac{ln\left(1-C_{i}{}^{\sigma_{i}\left(t\right)}\right)-ln\left(1-C_{i}{}^{-1}\right)}{b_{1i}lnC_{i}}+\frac{1}{b_{2i}\left(1-a_{i}\right)}$$

**Figure 1:**
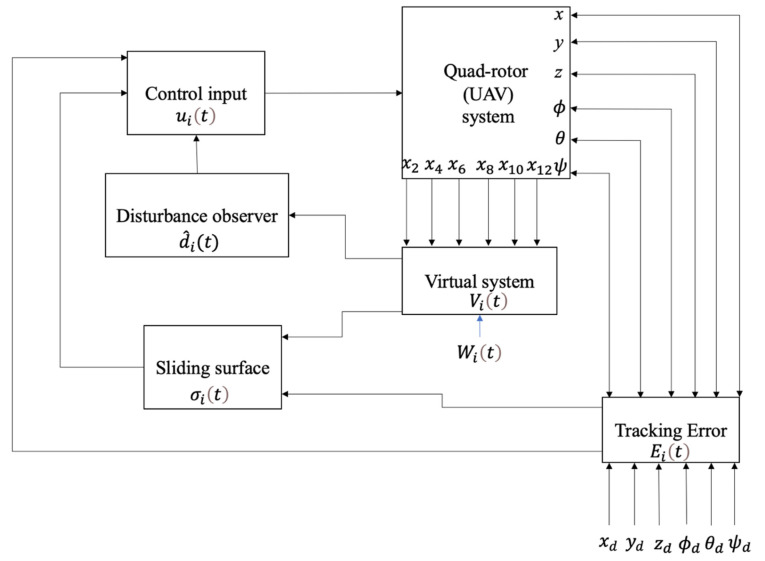
Block diagram of non-singular terminal SMC based on disturbance observer.

The block diagram of non-singular TSMC method based on the disturbance observer is illustrated in Figure 1.

As it is shown in the block diagram of the proposed control method, the variables of the quadrotor system were obtained based on the dynamical model. Then, the desired values are determined and the tracking errors *E_i_*(*t*) were obtained based on subtraction of actual and desired values of state trajectories. Afterward, the disturbance observer variables *V_i_*(*t*) were defined according to the supplementary variables *W_i_*(*t*) and quadrotor′s state variables. Now, with the usage of the disturbance observer variables and tracking errors, the sliding variables σ_*i*_(*t*) were defined. Moreover, the disturbance observer ${\hat{d}}_{i}\left(t\right)$ was gained by using the disturbance observer variable *V_i_*(*t*). Then, the control inputs *u_i_*(*t*) were obtained based on the disturbance observer and sliding variable and were entered to the quadrotor system. This control loop is repeated at any moment.

## 5. Simulation Results

In the subsequent section, simulation results of the quadrotor system using the proposed method presented in the previous sections are shown. Moreover, for the demonstration of the suggested method, simulation results were compared with the proposed method in [1]. The values of the parameters of quadrotor are given in Table 2.

**Figure 2:**
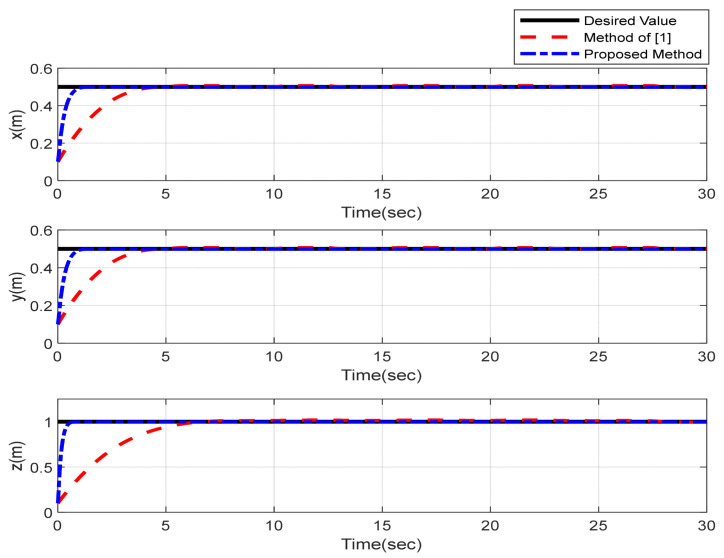
Position tracking of quadrotor using non-singular TSMC method.

**Figure 3:**
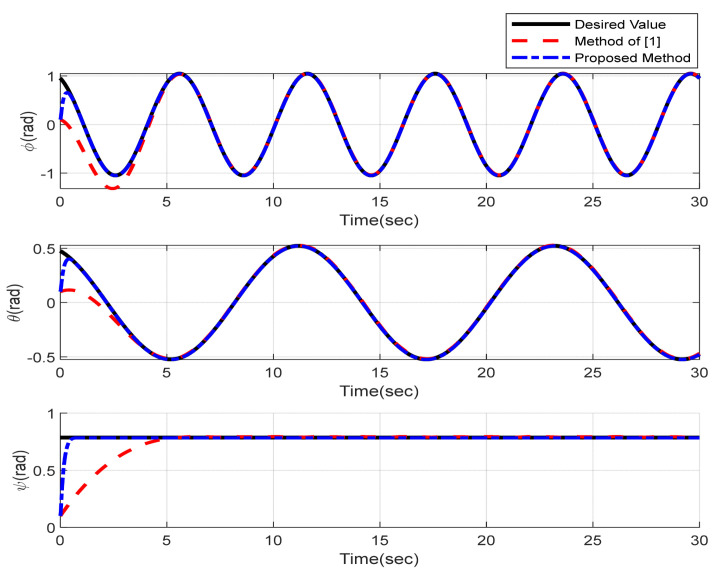
Attitude tracking of quadrotor using non-singular TSMC method.

**Figure 4:**
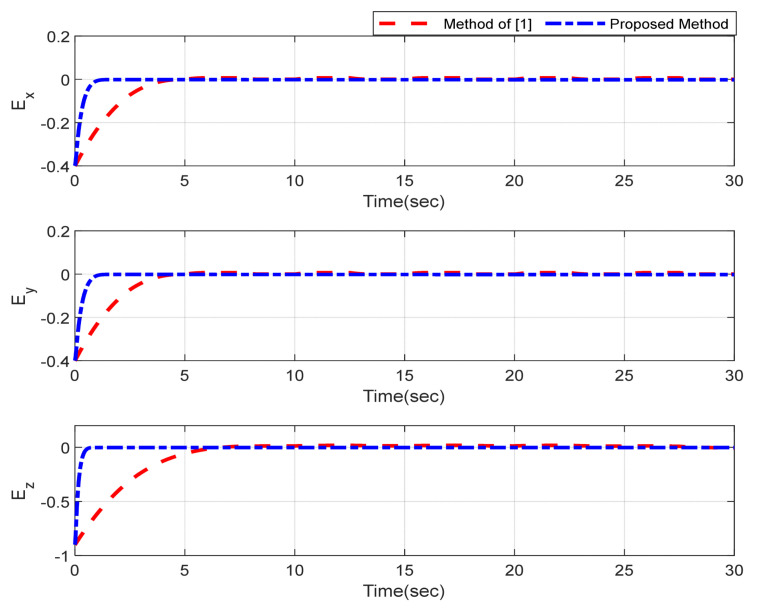
Trajectories of position tracking errors.

**Figure 5:**
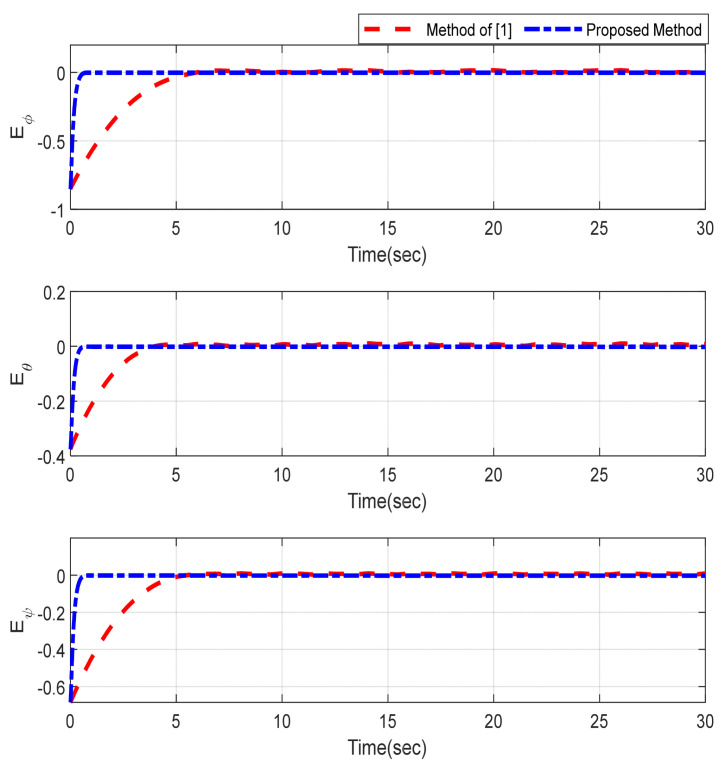
Trajectories of attitude tracking errors.

**Figure 6:**
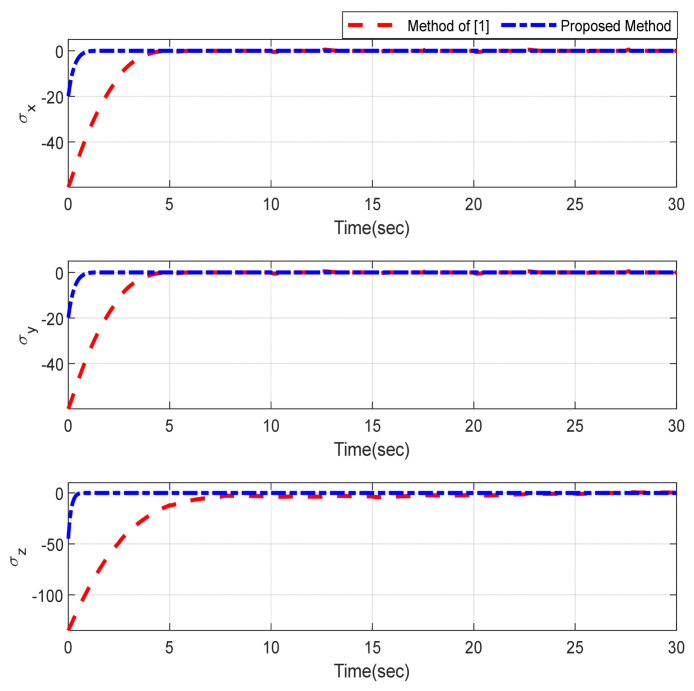
Sliding surfaces related to the position.

**Figure 7:**
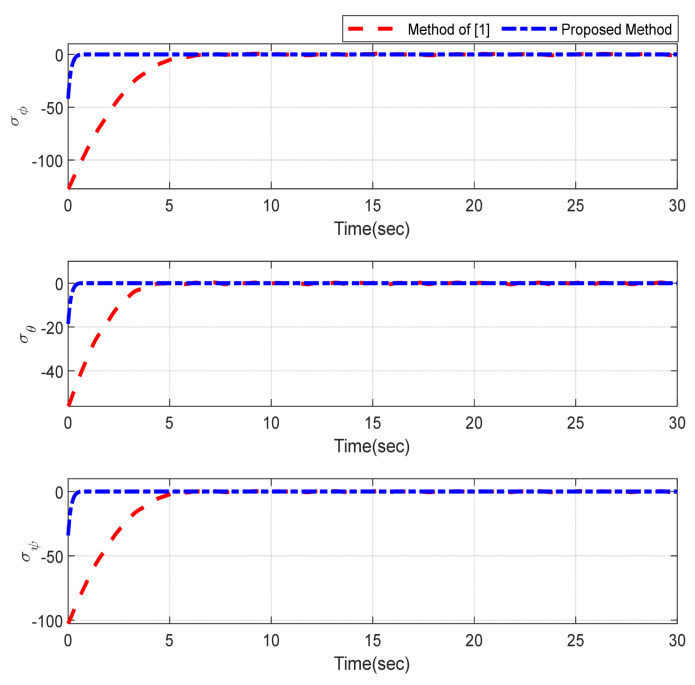
Sliding surfaces related to the attitude.

**Figure 8:**
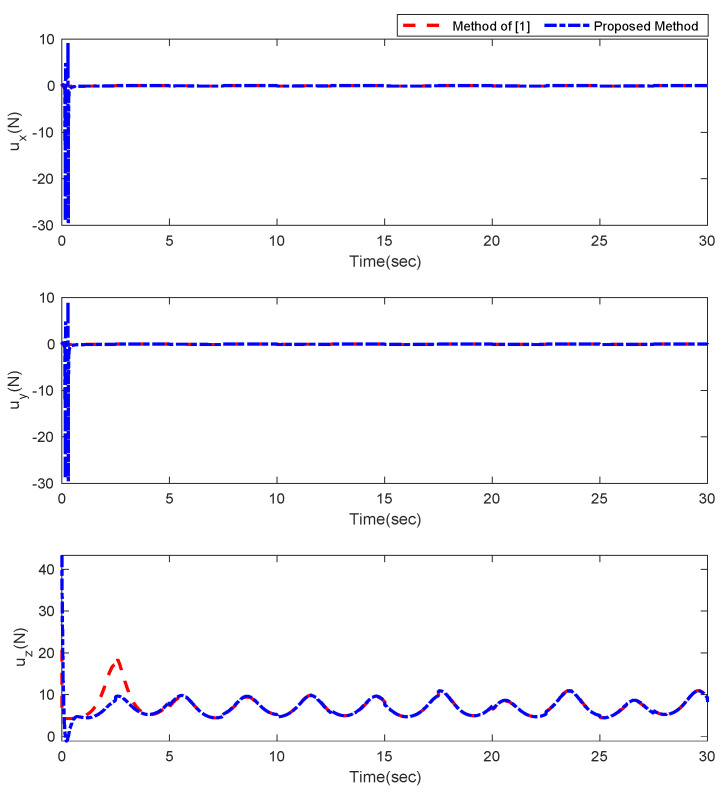
Control inputs relevant to position of quadrotor.

**Figure 9:**
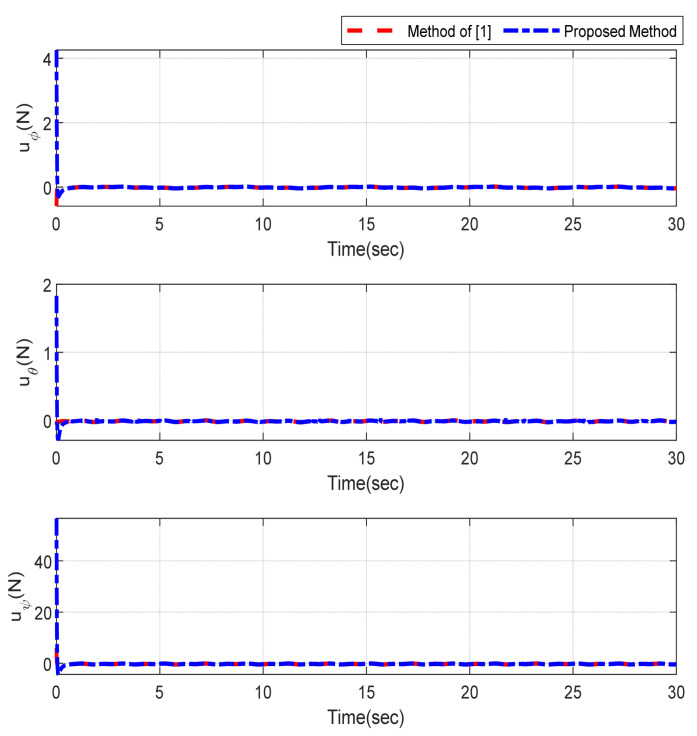
Control inputs relevant to attitude of quadrotor.

**Figure 10:**
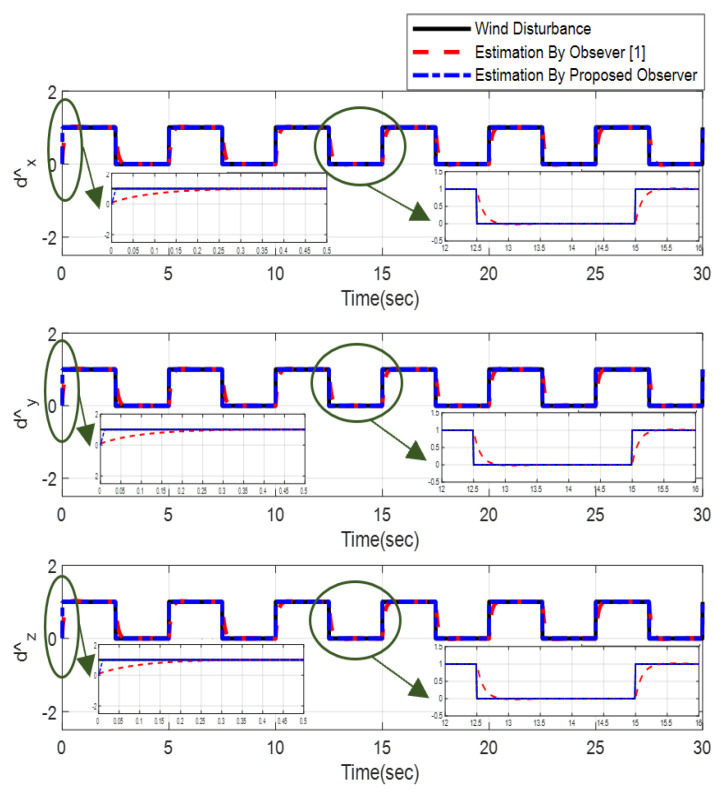
Estimation of the wind perturbation entered to the position of quadrotor.

**Figure 11:**
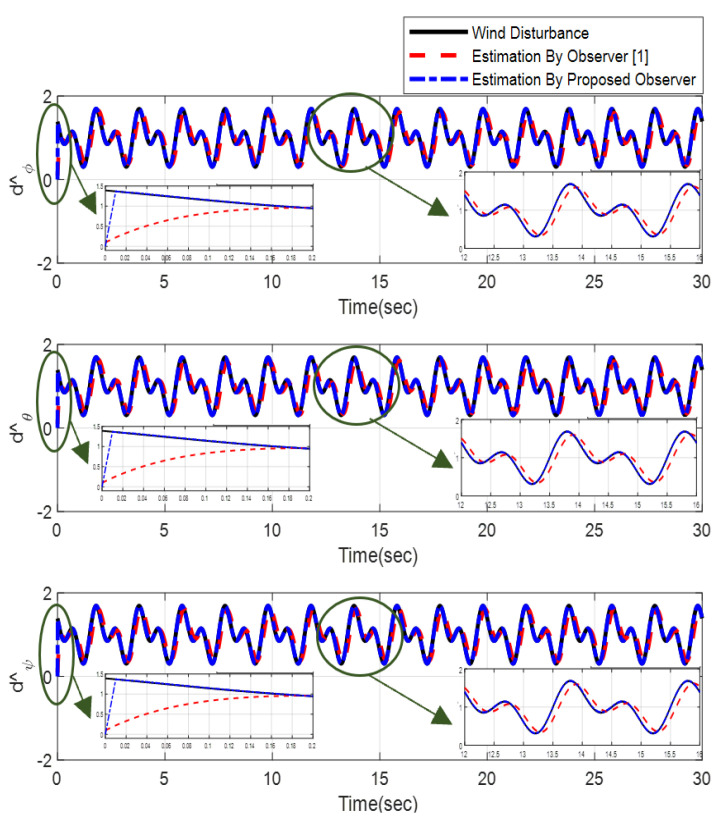
Estimation of wind perturbation entered to the attitude of quadrotor.

**Figure 12:**
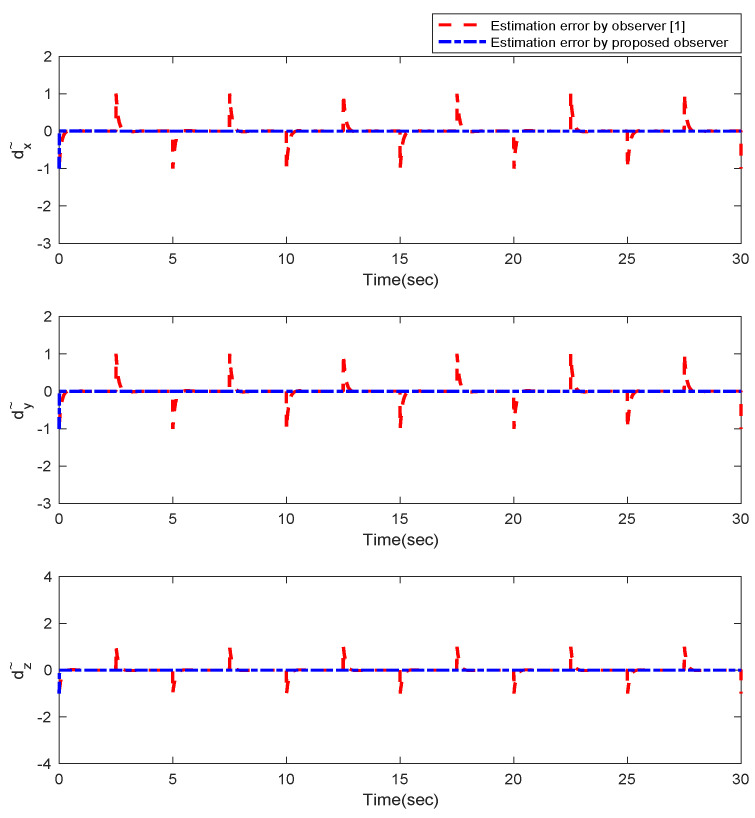
Estimation error of the observer for position of quadrotor.

**Figure 13:**
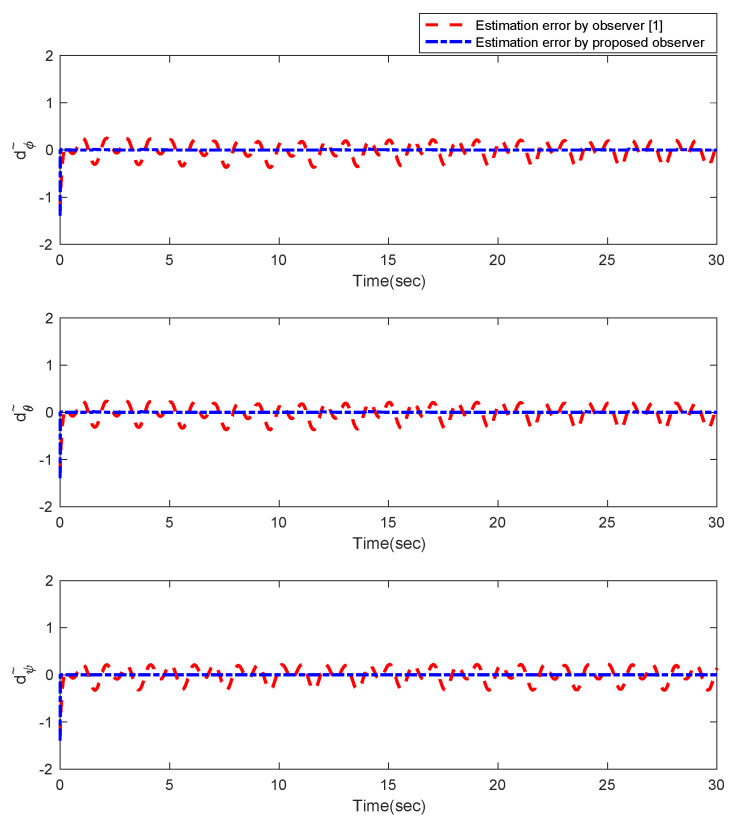
Estimation error of the observer for attitude of quadrotor.

Furthermore, the designing parameters which have been obtained by a trial-and-error method are given in Table 3. In Figure 2 and Figure 3, position and attitude tracking of the quadrotor are shown. Based on these figures, it can be observed that finite time tracking of position and attitude desired was performed properly and the proposed method showed faster tracking with respect to the method of [1]. Hence, time histories of the position and attitude tracking errors are displayed in Figure 4 and Figure 5 which confirms the finite time tracking using non-singular TSMC. Additionally, the reachability time of the proposed method was better that method of [1]. Time responses of sliding surfaces are illustrated in Figure 6 and Figure 7. From these figures, it can be stated that sliding surfaces based on the non-singular TSMC method converged to zero in finite-time and showed good performance compared with the recommended sliding surface in [1]. Control inputs which were obtained based on the non-singular TSMC method were exhibited and compared with the control input which was obtained using the method of [1] in Figure 8 and Figure 9. Estimation of the wind perturbations which were entered to the quadrotor system is depicted in Figure 10 and Figure 11. It can be concluded that wind perturbations are approximated in finite time. Moreover, transient and steady performance of the proposed observer was higher than the suggested observer in [1]. The estimation error of the proposed observer related to the position and attitude of the quadrotor are displayed in Figure 12 and Figure 13, respectively, and compared with the estimation error of the observer designed in [1]. It can be observed that the proposed observer owns better and faster transient and steady-states responses compared to the observer of [1].

According to these simulation outcomes, it can be inferred that, proposed non-singular terminal sliding surface (23) has better and faster performance respect to the suggested sliding surface in [1]. Furthermore, disturbance observer (8) can operate and estimated wind perturbations more better than suggested disturbance observer in [1]. All in all, the proficiency and success of proposed method is proved in comparison with method of [1].

## 6. Conclusions

In this paper, the dynamical model of the quadrotor was presented in position and attitude subsystems. A dynamical model of each subsystem was obtained under wind disturbances. The disturbance observer was designed for approximation of the wind perturbation. Afterward, with the target of position and attitude tracking control of the quadrotor in the existence of wind perturbation, the non-singular terminal sliding mode control method was offered. Additionally, with the usage of the Lyapunov stability theory, finite time reachability of the closed-loop position and attitude was acknowledged. Finally, the simulation and comparison results were provided to confirm the validity of the recommended method respect to other methods.

## Figures and Tables

**Table 1:** Parameters of dynamical model of quadrotor [33].

Variable	Unit	Name	Variable	Unit	Name
x, y, z	(m)	*Coordinate axes of quadrotor*	$I_{x},\;I_{y},\;I_{z}$	(N·m/rad/s^2^)	*Inertia to the axes * x, y, z
ϕ, θ, ψ	(Rad)	*Pitch, Roll, Yaw angles*	$C_{D}$	(N·m/rad/s)	*Drag factors*
$K_{fdx},\;K_{fdy},K_{fdz}$	(N/rad/s)	*Drag coefficients*	$J_{r}$	(N·m/rad/s^2^)	*Motor inertia*
$K_{fax},\;K_{fay},\;K_{faz}$	(N/rad/s)	*Aerodynamic fiction factors*	$K_{p}$	(N·m/rad/s)	*Lift power facto*
m	(kg)	*Mass of quadrotor*	$w_{1}$ *,* $w_{2}$ *,* $w_{3}$ *,* $w_{4}$	(Rad/s)	*Angular velocities*
d	(m)	*Distance between rotation axes and center*			

**Table 2:** Parameters of the quadrotor’s system [33].

Variable (Unit)	Quantity	Variable (Unit)	Quantity
*m* (kg)	0.486	$K_{fdy\;}\left(N/\mathrm{rad}/s\right)$	5.5670 × 10^−4^
*d* (m)	0.25	$I_{x}\;$(N·m/rad/s^2^)	3.8278 × 10^−3^
*C_d_* (N·m/rad/s)	3.2320 × 10^−2^	$I_{y}\;$(N·m/rad/s^2^)	3.8278 × 10^−3^
$J_{r}\;$(N·m/rad/s^2^)	2.8385 × 10^−5^	$I_{z}\;$(N·m/rad/s^2^)	7.6566 × 10^−3^
$K_{fay}\;\left(N/\mathrm{rad}/s\right)$	5.5670 × 10^−4^	$K_{fdz\;}\left(N/\mathrm{rad}/s\right)$	6.3540 × 10^−4^
$K_{faz}\;\left(N/\mathrm{rad}/s\right)$	6.3540 × 10^−4^	$K_{p}\;\left(N\cdot m/\mathrm{rad}/s\right)$	2.9842 × 10^−3^
$K_{fdx}\;\left(N/\mathrm{rad}/s\right)$	5.5670 × 10^−4^	$K_{fax}\;\left(N/\mathrm{rad}/s\right)$	5.5670 × 10^−4^

**Table 3:** Parameters of the control strategy,∀i=x,y,z,ϕ,θ,ψ.

**Variable**	Quantity	Variable	Quantity
$\left[x_{d}\left(t\right),y_{d}\left(t\right),z_{d}\left(t\right)\right]$	$\left[0.5,0.5,1\right]$	$m_{i}$	9
$\phi_{d}\left(t\right)$	$\frac{\pi }{3}\sin\left(\frac{\pi }{3}+t\right)$	$\gamma_{i}$	0.1
$\theta_{d}\left(t\right)$	$\frac{\pi }{6}\sin\left(\frac{\pi }{6}+t\right)$	$a_{i}$	3/5
$\psi_{d}\left(t\right)$	π/4	$n_{i}$	7
$\ell_{i},\;$	50	$d_{i}\left(t\right),\forall i=\phi ,\theta ,\psi$	$1-0.39\sin\left(2\pi t\right)+0.39\cos\left(\pi t\right)$
$k_{i}$	2	$d_{i}\left(t\right)\;,\forall i=x,y,z$	Pulse generator(Pulse width: 50, period: 5, amplitude: 1)
$\varepsilon_{i},$	0.1	$x_{i}\left(0\right)\;,\;\forall i=1,\ldots ,\;12$	0.1
$\eta_{1},\eta_{2}$	7/9	$b_{1,i}$	500

## Data Availability

The data that support the findings of this study are available within the article.
